# Full Toxicity Assessment of Genkwa Flos and the Underlying Mechanism in Nematode *Caenorhabditis elegans*

**DOI:** 10.1371/journal.pone.0091825

**Published:** 2014-03-13

**Authors:** Yan Qiao, Yunli Zhao, Qiuli Wu, Lingmei Sun, Qinli Ruan, Yanyan Chen, Meng Wang, Jinao Duan, Dayong Wang

**Affiliations:** 1 Jiangsu Key Laboratory for High Technology Research of TCM Formulae, Nanjing University of Chinese Medicine, Nanjing, China; 2 Key Laboratory of Developmental Genes and Human Disease in Ministry of Education, Medical School of Southeast University, Nanjing, China; CSIR-Central Drug Research Institute, India

## Abstract

Genkwa Flos (GF), the dried flower bud from *Daphne genkwa* Sieb. et Zucc. (Thymelaeaceae), is a well-known and widely used traditional Chinese medicine. However, we know little about the *in vivo* mechanism of GF toxicity. Nematode *Caenorhabditis elegans* has been considered as a useful toxicity assay system by offering a system best suited for asking the *in vivo* questions. In the present study, we employed the prolonged exposure assay system of *C. elegans* to perform the full *in vivo* toxicity assessment of raw-processed GF. Our data show that GF exposure could induce the toxicity on lifespan, development, reproduction, and locomotion behavior. GF exposure not only decreased body length but also induced the formation of abnormal vulva. The decrease in brood size in GF exposed nematodes appeared mainly at day-1 during the development of adult nematodes. The decrease of locomotion behavior in GF exposed nematodes might be due to the damage on development of D-type GABAergic motor neurons. Moreover, we observed the induction of intestinal reactive oxygen species (ROS) production and alteration of expression patterns of genes required for development of apical domain, microvilli, and apical junction of intestine in GF exposed nematodes, implying the possible dysfunction of the primary targeted organ. In addition, GF exposure induced increase in defecation cycle length and deficits in development of AVL and DVB neurons controlling the defecation behavior. Therefore, our study implies the usefulness of *C. elegans* assay system for toxicity assessment from a certain Chinese medicine or plant extract. The observed toxicity of GF might be the combinational effects of oxidative stress, dysfunction of intestine, and altered defecation behavior in nematodes.

## Introduction

Genkwa Flos (GF), the dried flower bud from *Daphne genkwa* Sieb. et Zucc. (Thymelaeaceae), is widely distributed in the regions of Yangtze River and Yellow River in China. GF is a well-known traditional Chinese medicine (TCM). GF has been used for the purposes of diuretic, antitussive, expectorant, abortifacient, and antitumor for centuries [1]. In GF, a series of compounds including flavonoids and daohne diterpene esters have been identified and isolated [2]–[3]. GF or compounds from GF can be potentially used for anti-inflammation, anti-complement, anti-melanogenesis, anti-virus, and anti-leukemia [4]–[8]. Moreover, because *Daphne genkwa* plants can be used as the effective remedy to treat various tumors [9], some compounds have also been isolated from *Daphne genkwa* plants because of their antitumor activity [10]–[15].

It has been commonly recognized that *Daphne genkwa* is a toxic shrub [15]. Previous study has investigated the cytotoxicity of compounds from *Daphne genkwa* with the aid of cancer cell lines [16]. The excessive or chronic application of GF also resulted in the hepatotoxicity on animals [17]–[18]. Recently, the toxicity of daphnane-type diterpenoids extracted from GF was further examined in rat with the aid of mortality and time to death as the toxicity assessment endpoints [19]. However, so far the toxicity information on GF is still very limited. Especially, we still know little about the *in vivo* mechanism for GF toxicity.

Free-living nematode *Caenorhabditis elegans*, a thoroughly studied model animal, has been considered as a useful alternative toxicity assay system for mammalians [20]–[22]. The experimental potential of *C. elegans* can offer a system best suited for asking the *in vivo* questions [20]–[22]. *C. elegans* has been used in both toxicity assessment and toxicological study for many toxicants including metals [23]–[36], organic pollutants [37]–[42], and engineered nanomaterials (ENMs) [43]–[53]. It has been proven that *C. elegans* can be further used for toxicological study of drugs [54]–[58]. Especially, several studies have been performed on the investigations on beneficial or adverse effects of components isolated from specific plants and beneficial effects of specific Chinese medicines with the aid of *C. elegans* as the assay system [59]–[66].

In the present study, we employed the *C. elegans* assay system to perform the full *in vivo* toxicity assessment of raw-processed GF. Moreover, we tried to examine the possible underlying mechanism for the GF toxicity in nematodes. Our data here will be helpful for our understanding the possible underlying *in vivo* mechanism of GF toxicity. In addition, our study further implies the usefulness of *C. elegans* assay system for the assessment of adverse effects from a certain Chinese medicine or plant extract.

## Results

### GF Exposure at the Examined Concentrations did not Induce Lethality of Nematodes

Considering the property of long-term application for Chinese medicines, we performed the prolonged exposure for toxicity assessment of GF in nematodes. Prolonged exposure was performed from L1-larvae to young adult (Fig. S1A). Concentrations selected for GF exposure were 0.12, 0.14, 0.16, 0.18, 0.2, 0.22, and 0.24 g/mL (Fig. S1B). After prolonged exposure to the examined concentrations of GF, we did not observe the lethality of nematodes (Fig. S1B), implying that GF at the examined concentrations may be not lethal to the exposed organisms.

### Effects of GF Exposure on Lifespan of Nematodes

**Figure 1:**
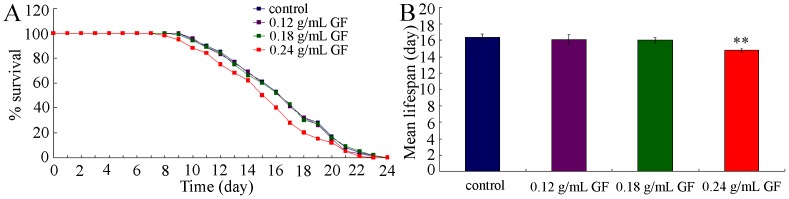
Comparison of lifespan in nematodes exposed to GF. (A) Lifespan curves of nematodes exposed to GF. (B) Comparison of mean lifespans in nematodes exposed to GF. Exposures were performed from L1-larvae to young adult. GF, Genkwa Flos. Bars represent means ± S.E.M. **p<0.01.

We further investigated the effects of GF exposure on lifespan of nematodes. Lifespan is an important endpoint to reflect the long-term effects of a specific toxicant. Prolonged exposure to 0.12–0.18 g/mL of GF did not significantly alter lifespan of nematodes (Fig. 1). In contrast, prolonged exposure to 0.24 g/mL of GF significantly reduced the lifespan of nematodes (Fig. 1).

### Effects of GF Exposure on Development of Nematodes

**Figure 2:**
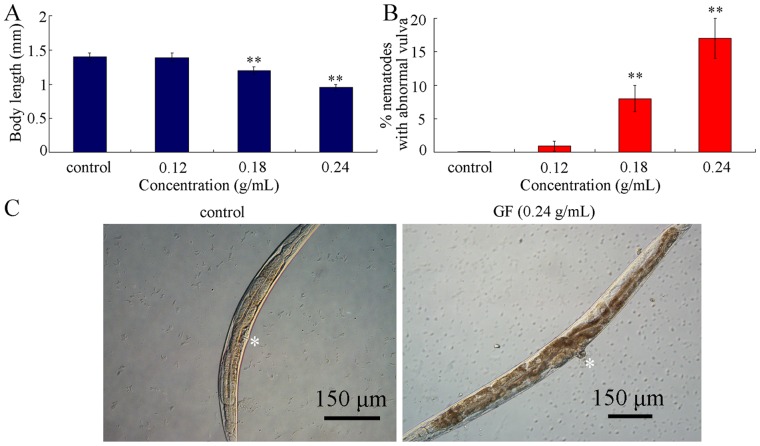
Effects of prolonged exposure to GF on development of nematodes. (A) Effects of GF exposure on body length. (B) Effects of GF exposure on percentage of nematodes with abnormal vulva. (C) Pictures showing the nematodes with abnormal vulva induced by GF. Asterisks show the positions of vulva. GF, Genkwa Flos. Exposures were performed from L1-larvae to young adult. Bars represent means ± S.E.M. **p<0.01.

To examine the effects of prolonged exposure to GF on development, we first investigated the body length in control and GF-exposed nematodes. Although prolonged exposure to 0.12 g/mL of GF did not noticeably influence the body length of nematodes, prolonged exposure to 0.18–0.24 g/mL of GF significantly reduced the body length of nematodes (Fig. 2A). Moreover, we observed that prolonged exposure to 0.18–0.24 g/mL of GF significantly induced the formation of abnormal vulva (Fig. 2B). Prolonged exposure to 0.12 g/mL of GF did not obviously affect the morphology of exposed nematodes (Fig. 2B). Compared with the control vulva having a slightly smooth surface, the abnormal vulva induced by GF exposure had a large protuberance (Fig. 2C).

### Effects of GF Exposure on Reproduction of Nematodes

**Figure 3:**
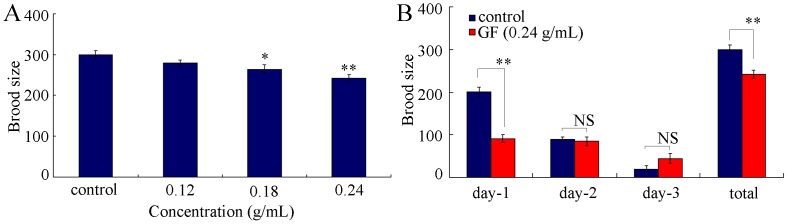
Effects of prolonged exposure to GF on reproduction of nematodes. (A) Effects of GF exposure on brood size of nematodes. (B) Effects of GF exposure on brood size of day-1, day-2 and day-3 adult nematodes. GF, Genkwa Flos. Exposures were performed from L1-larvae to young adult. Bars represent means ± S.E.M. NS, no significance. *p<0.05, **p<0.01.

In nematodes, reproductive organ is one of the important secondary targeted organs for toxicants [22], [67]–[69]. We further investigated the effects of prolonged exposure to GF on reproduction with the aid of brood size as the endpoint. Prolonged exposure to 0.12 g/mL of GF did not obviously influence the brood size of nematodes (Fig. 3A). In contrast, prolonged exposure to 0.18–0.24 g/mL of GF significantly reduced the brood size of nematodes (Fig. 3A). More interestingly, the decrease in brood size in GF exposed nematodes appeared mainly at day-1 during the development of adult nematodes (Fig. 3B). We did not observe the significant differences of brood size at day-2 or day-3 during the development of adult nematodes between control and GF exposure (Fig. 3B).

### Effects of GF Exposure on Locomotion Behavior of Nematodes

**Figure 4:**
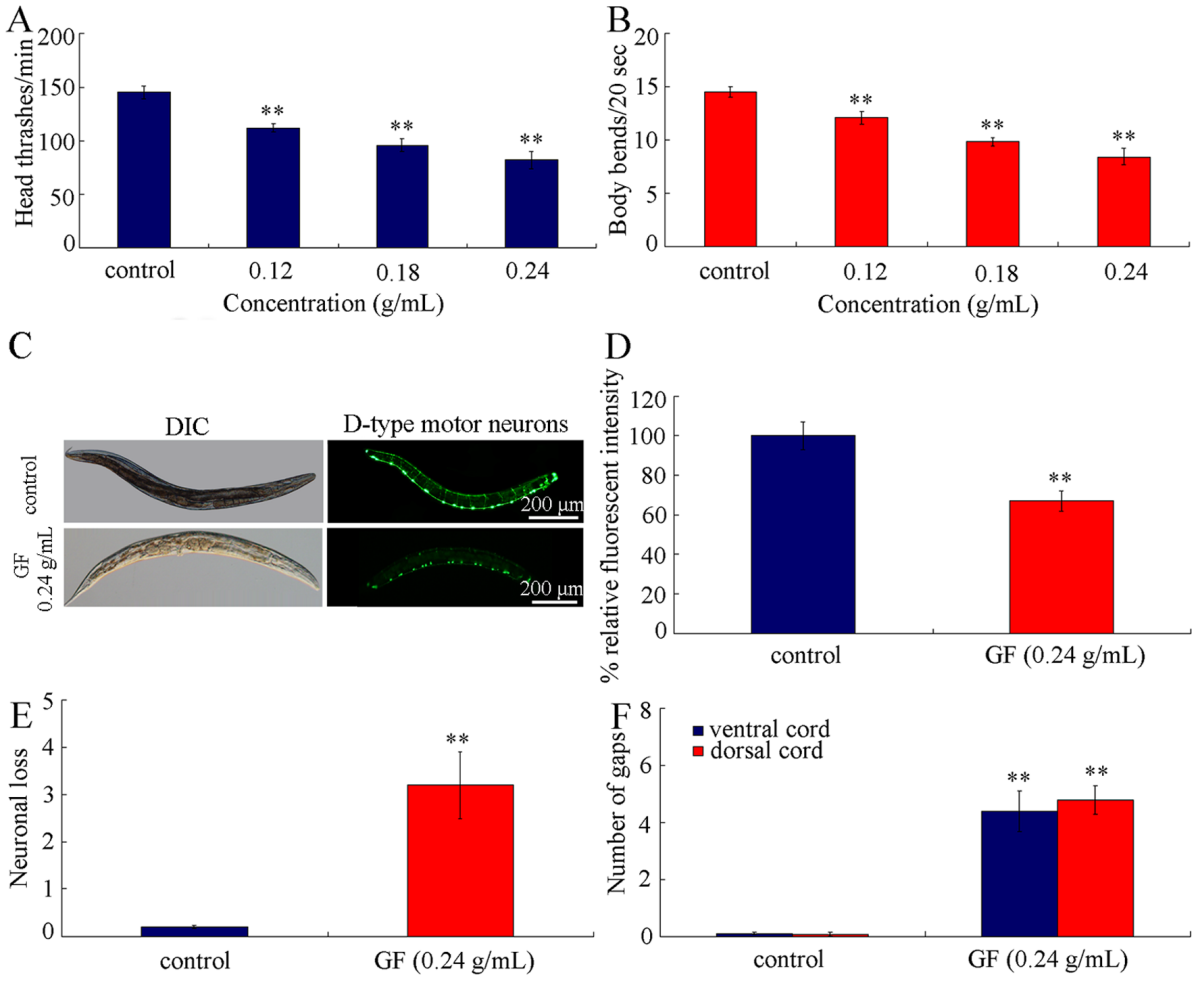
Effects of prolonged exposure to GF on locomotion behavior and development of D-type GABAergic motor neurons in nematodes. (A) Effects of GF exposure on head thrash. (B) Effects of GF exposure on body bend. (C) Effects of GF exposure on development of D-type GABAergic motor neurons. (D) Effects of GF exposure on relative fluorescent intensity of cell bodies at ventral cord of D-type GABAergic motor neurons. (E) Effects of GF exposure on neuronal loss of D-type GABAergic motor neurons. (F) Effects of GF exposure on gap formation on ventral or dorsal cord of D-type GABAergic motor neurons in nematodes. GF, Genkwa Flos. Exposures were performed from L1-larvae to young adult. Bars represent means ± S.E.M. **p<0.01.

In nematodes, neuron may be another secondary targeted organ for toxicants [22], [42], [51], [70]. Previous studies have demonstrated that locomotion behavior is a relatively sensitive endpoint for toxicity assessment in nematodes [36], [48], [53]. We next investigated the effects of prolonged exposure to GF on locomotion behavior in nematodes. Prolonged exposure to all the examined concentrations of GF significantly decreased both head thrash and body bend of nematodes (Figs. 4A and 4B). Our data here further suggest the sensitivity of locomotion behavior for toxicity assessment in nematodes.

### GF Exposure Affects the Development of D-type GABAergic Motor Neurons in Nematodes

In *C. elegans*, locomotion behavior is under the control of D-type GABAergic motor neurons [71]. Prolonged exposure to 0.24 g/mL of GF obviously influenced the development of D-type GABAergic motor neurons (Fig. 4C). The severe deficits in axon development were observed in 0.24 g/mL of GF exposed nematodes (Fig. 4C). Prolonged exposure to 0.24 g/mL of GF significantly decreased the fluorescent intensity of cell bodies for ventral cord D-type GABAergic motor neurons (Fig. 4D). Moreover, prolonged exposure to 0.24 g/mL of GF noticeably induced the neuronal loss and the gap formation on both ventral and dorsal cords of D-type GABAergic motor neurons (Figs. 4E and 4F). Thus, GF exposure can potentially cause damage on development of D-type GABAergic motor neurons in nematodes.

### Effects of GF Exposure on Intestinal Reactive Oxygen Species (ROS) Production in Nematodes

**Figure 5:**
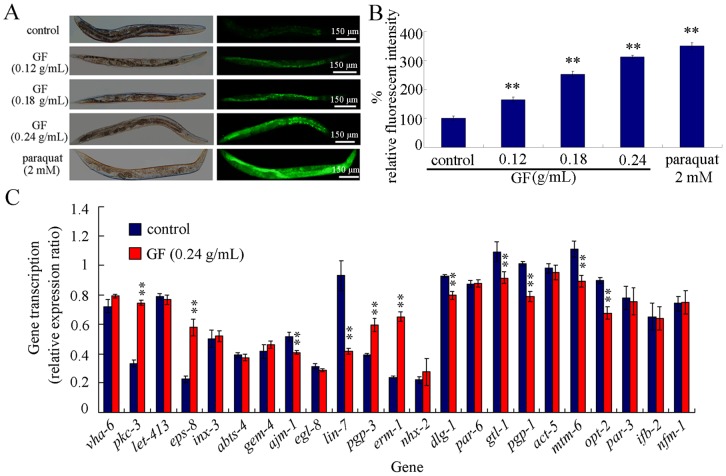
Effects of prolonged exposure to GF on intestinal development in nematodes. (A) Pictures showing the effects of GF exposure on intestinal ROS production. Paraquat (2 mM) treatment for 12-hr from L4-larvae was used as a positive control. Paraquat is a commonly used ROS-generator. (B) Comparison of intestinal ROS production between control and GF exposed nematodes. (C) Expression patterns of genes required for intestinal development in control and GF exposed nematodes. The results were expressed as the relative expression ratio between the targeted gene and the reference *tba-1* gene. GF, Genkwa Flos. Exposures were performed from L1-larvae to young adult. Bars represent means ± S.E.M. **p<0.01.

In nematodes, intestine is the primary targeted organ for toxicants [22], [46], [67]–[69], [72]. Previous studies have implied that the functional state of intestinal barrier is required for nematodes to protect themselves from the damage of toxicants [67]–[69]. To examine the possible mechanism explaining the GF toxicity, we investigated the effects of prolonged exposure to GF on intestinal ROS production. After prolonged exposure, GF at concentrations of 0.12–0.24 g/mL significantly induced the intestinal ROS production (Figs. 5A and 5B), implying the damage from GF exposure on intestinal development in nematodes.

### Alteration of Expression Patterns of Genes Required for Intestinal Development in Nematodes

Moreover, we found that prolonged exposure to 0.24 g/mL of GF significantly altered the expression patterns of some genes required for intestinal development in nematodes [73]. Although GF exposure did not influence the expression patterns of *vha-6*, *let-413*, *inx-3*, *abts-4*, *gem-4*, *egl-8*, *nhx-2*, *par-6*, *act-5*, *par-3*, *ifb-2*, and *nfm-1* genes, GF exposure significantly increased expression levels of *pkc-3*, *eps-8*, *pgp-3*, and *erm-1* genes and decreased expression levels of *ajm-1*, *lin-7*, *dlg-1*, *gtl-1*, *pgp-1*, *mtm-6*, and *opt-2* genes (Fig. 5C, Fig. S2). In *C. elegans*, *pkc-3* gene encodes an atypical protein kinase, *eps-8* gene encodes a homolog of mouse epidermal growth factor receptor kinase substrate, *pgp-1* and *pgp-3* genes encode transmembrane protein, *erm-1* gene encodes an ortholog of the ERM family of cytoskeletal linker, *ajm-1* gene encodes a member of the apical junction molecule class, *lin-7* gene encodes a protein containing a PDZ domain and an L27 domain, *dlg-1* gene encodes a MAGUK protein, *gtl-1* gene encodes a TRPM subfamily member of the TRP channel family, *mtm-6* gene encodes a myotubularin lipid phosphatase orthologous, and *opt-2* gene encodes a high-affinity, proton-coupled oligopeptide transporter (Table S1).

### Effects of GF Exposure on Defecation Behavior in Nematodes

**Figure 6:**
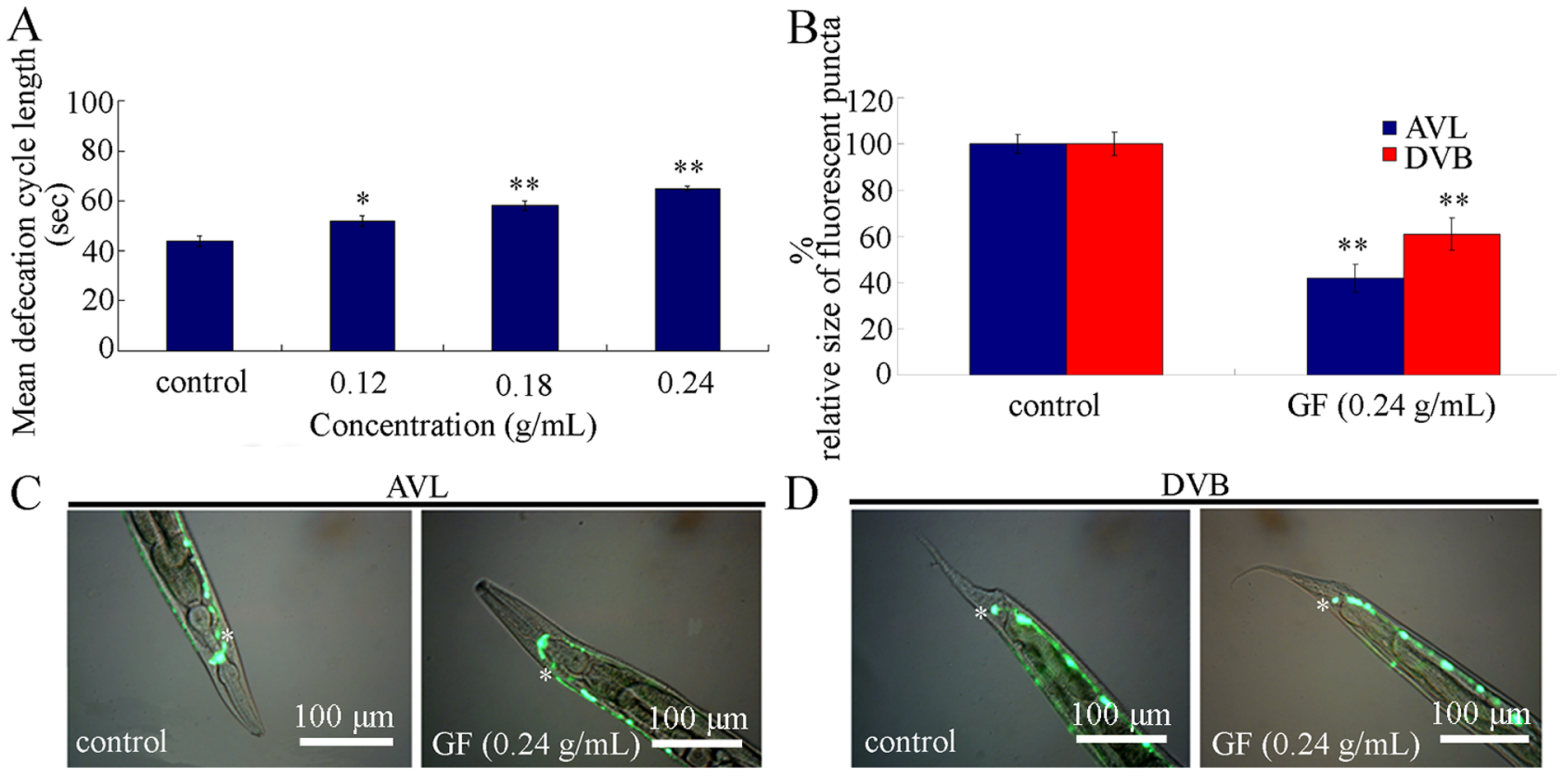
Effects of prolonged exposure to GF on defecation behavior and development of neurons controlling defecation behavior in nematodes. (A) Effects of GF exposure on mean defecation cycle length. (B) Effects of GF exposure on relative size of fluorescent puncta of AVL or DVB neurons. (C) Effects of GF exposure on development of AVL neurons. (D) Effects of GF exposure on development of DVB neurons. GF, Genkwa Flos. Exposures were performed from L1-larvae to young adult. Bars represent means ± S.E.M. *p<0.05, **p<0.01.

Besides the intestinal development, defecation behavior may also be an important regulator for toxicity formation of toxicants in nematodes [68]. We further investigated the effects of prolonged exposure to GF on defecation behavior with the aid of mean defecation cycle length as the assay endpoint. After prolonged exposure, GF at concentrations of 0.12–0.24 g/mL significantly increased the mean defecation cycle length (Fig. 6A), which implies the possible damage of GF on structures controlling defecation behavior or the difficulty of toxic components of GF to be excreted out of the body.

### Damage of GF Exposure on Development of Neurons Controlling Defecation Behavior in Nematodes

In nematodes, the defecation behavior is controlled by both AVL neuron in head and DVB neuron in tail [52]. To determine whether GF exposure would cause damage on structures controlling defecation behavior, we examined the effects of prolonged exposure to GF on development of AVL and DVB neurons. After prolonged exposure, GF at the concentration of 0.24 g/mL significantly decreased the relative size of fluorescent puncta for both AVL neurons and DVB neurons in nematodes (Figs. 6B–6D). These results suggest that prolonged exposure to GF can result in the damage on neurons controlling the defecation behavior.

### Molecular Basis for GF to Reduce the Lifespan in Nematodes

**Figure 7:**
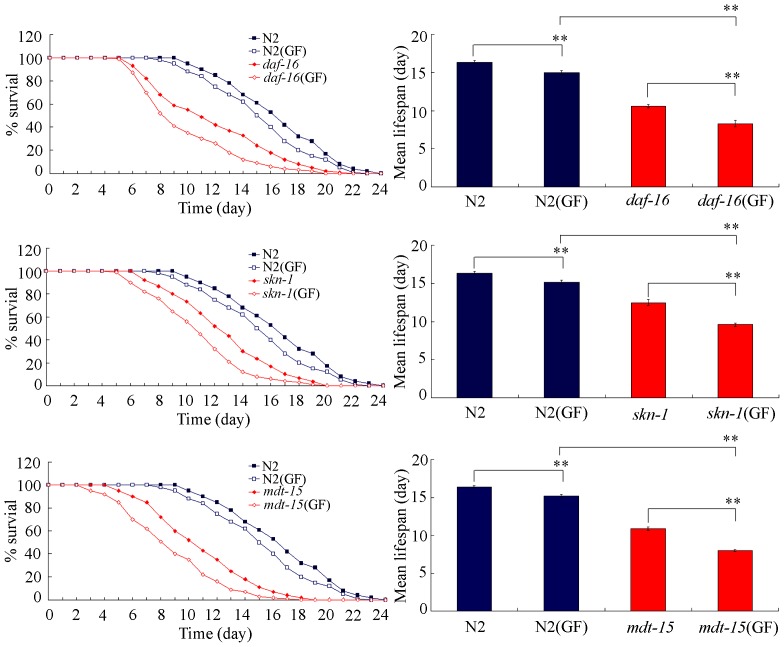
Comparison of lifespan in wild-type and mutant nematodes exposed to GF. GF, Genkwa Flos. Exposures were performed from L1-larvae to young adult. Bars represent means ± S.E.M. **p<0.01.

To determine whether certain genes playing key roles in detoxification are required for the control of GF toxicity, we examined the effects of mutations of *daf-16* [74], *skn-1* [75], or *mdt-15* [76] gene on GF toxicity formation in nematodes. With the aid of lifespan as the endpoint, interestingly, we found that the *daf-16(mu86)*, *skn-1(zu67)*, and *mdt-15(tm2182)* mutants had the susceptible property to the GF toxicity (Fig. 7). These data confirm the possible involvement of *daf-16*, *skn-1* and *mdt-15* mediated signaling pathways in regulating the GF toxicity formation.

### Identification of Chemical Components in Raw-processed GF

**Figure 8:**
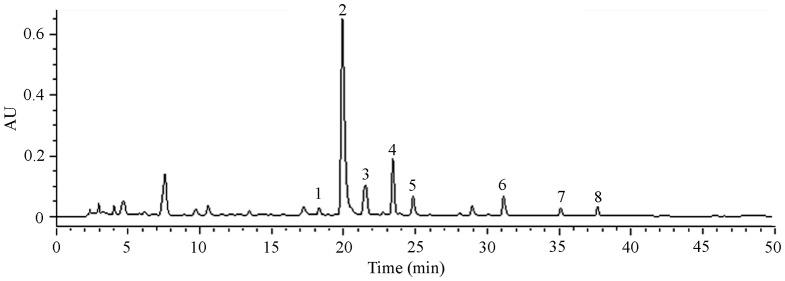
Chemical components in GF analyzed by HPLC. 1, luteolin-5-O-β-D-glucoside; 2, apigenin-5-O-β-D-glucoside, 3, genkwanin-5-O-β-D-primeveroside; 4, genkwanin-5-O-β-D-glucoside; 5, genkwanin-4′-O-β-D*-* lutinoside; 6, apigenin; 7, 3′-hydroxygenkwanin; 8, genkwanin. GF, Genkwa Flos.

Using the reverse phase high-performance liquid chromatography (RP-HPLC) method, we finally identified the possible chemical components in our prepared GF solution. Based on the chromatographic fingerprint, the prepared GF solution contained the chemical components of luteolin-5-O-β-D-glucoside, apigenin-5-O-β-D-glucoside, genkwanin-5-O-β-D-primeveroside, genkwanin-5-O-β-D-glucoside, genkwanin-4′-O-β-D- lutinoside, apigenin, 3′-hydroxygenkwanin, and genkwanin, and some other still unknown compounds (Fig. 8).

## Discussion

Previous studies have demonstrated that *C. elegans* can be used for toxicity assessment of metals [23]–[36], organic pollutants [37]–[42], ENMs [43]–[53], or specific components extracted from plants [62]. In this study, our data further imply that *C. elegans* can be used for toxicity assessment of specific Chinese medicines or plant extracts. Thus, *C. elegans* can be used for evaluation of both the beneficial effects [59]–[61], [63]–[66] and the adverse effects of specific Chinese medicines or plant extracts. Because most of the Chinese medicines or plant extracts still lack the detailed toxicity data, *C. elegans* will serve as a rapid and systematic assay system for toxicity assessment of specific Chinese medicines or plant extracts.

In the present study, we performed the full assessment of GF toxicity utilizing the *in vivo C. elegans* assay system. Considering the property of long-term application for GF in clinical, we performed the prolonged exposure from L1-larvae to young adult for toxicity assessment of GF. L1-larvae may be more sensitive than adults for toxicity evaluation in nematodes [25], [77]. Our data demonstrate that prolonged exposure to GF could induce the toxicity on lifespan, development, reproduction and locomotion behavior (Figs. 1–4). Especially, GF exposure induced the formation of abnormal vulva (Fig. 2C). Such a deficit was also observed in heavy metal exposed nematodes [33], [78], which suggests that the induction of abnormal vulva may be a common phenotype for adverse effects of toxicants on nematodes.

For the mechanism explaining the GF toxicity, we raised several possibilities. One of the possibilities is the induction of structural deficits caused by GF exposure. For the decrease in locomotion behavior in GF exposed nematodes, we found that prolonged exposure to GF caused the damage on development of D-type GABAergic motor neurons (Figs. 4C–4F), which specially function in the control of locomotion behavior. The second possibility is that prolonged exposure to GF may result in the damage on intestinal development. We observed the severe induction of intestinal ROS production (Figs. 5A–5B) and alteration of expression patterns of some genes required for the intestinal development (Fig. 5C). In *C. elegans*, *mtm-6*, *pkc-3*, *pgp-1*, *opt-2*, *pgp-3*, and *gtl-1* genes are required for the development of apical domain of intestine, *erm-1* and *eps-8* are required for the microvilli development, and *ajm-1*, *dlg-1*, and *lin-7* genes are required for the development of apical junction of intestine [73]. These data suggest that GF exposure may at least result in the damage on development of apical domain, microvilli, and apical junction of intestine in nematodes. In addition, these data may also imply that the response of intestinal development and function may serve as an important physiological basis for the possible adaptation of nematodes to GF. The third possibility is that GF exposure induced the increase in mean defecation cycle length, which may in turn cause the deficits in AVL and DVB neurons controlling defecation behavior and the difficulty of excretion of GF out of the body in nematodes. Moreover, the phenotypic analysis of *daf-16*, *skn-1*, and *mdt-15* mutants suggests that at least *daf-16*, *skn-1*, and *mdt-15* mediated signaling pathways may function as the important molecular basis for the control of GF toxicity in nematodes. Therefore, the toxicity of GF is possibly the combinational effects of oxidative stress, dysfunction of intestine, and altered defecation behavior.

Our data here suggest the potential adverse effects of GF on nematodes after prolonged exposure. These results will be helpful for our understanding and attention of the careful use of this drug in clinical. At least based on our data, the duration, dose, and developmental stage of patients should be carefully considered before use of this drug. Meanwhile, some strategies, such as modulation of the processing procedure, can be also designed and considered to reduce the possible adverse effects from GF.

In the present study, the raw-processed GF was extracted after boiling treatment. During this extraction process, some possible thermolabile molecules having the bioactivity may be broken down. Therefore, the data provided here may be not able to reflect the possible biological effects of those possible thermolabile molecules in GF. Moreover, the GF can be extracted with different strategies. Besides the water boiling, the ethanolic extraction can also be used for GF preparation. The chemical components in raw-processed GF solutions prepared with different extraction strategies may be somewhat different, which implies that the obtained data here may be not able to completely reflect the biological effects of raw-processed GF solutions prepared with the other extraction strategies on nematodes.

In conclusion, with the Chinese medicine of GF as a sample, our data here provide the evidence to indicate the value of *C. elegans* assay system for the *in vivo* toxicity assessment of specific Chinese medicines or plant extracts. After prolonged exposure, GF induced multiple toxicities on nematodes, which may be due to the combinational effects of several mechanisms including oxidative stress, intestinal dysfunction, and abnormal defecation behavior. In the prepared GF solution, many chemical components may exist. In the future, examination of the beneficial or adverse effects of specific chemical components in GF will be further helpful for our understanding and controlling the toxicity formation and the clinical use of this drug.

## Materials and Methods

### Plant Materials and Chemicals

The dried flower buds of *Daphne* genkwa Sieb et Zucc. were collected from Liuan city, Anhui province, China. No specific permissions were required for the location/activity to collect the plants, because the location does not belong to any national park or protected area of land or sea. We also confirm that the field did not involve endangered behavior to the protected species. The flower buds were authenticated, and the voucher specimen (No. 110326) has been deposited at the Herbarium in Jiangsu Key Laboratory for High Technology Research of Traditional Chinese Medicine Formulae, Nanjing University of Chinese Medicine. The raw-processed GF (24 g) was decocted 2 times with 240 mL of boiling distilled water for 2-hr. The decocted suspensions were filtered, collected, and concentrated by decompressive rotary evaporation. The prepared solutions were stored at −20°C before use. The prepared raw-processed GF can be directly used in the clinical. All the other chemicals were obtained from Sigma-Aldrich (St. Louis, MO, USA).

### Strain Preparation

Nematodes used in the present study were wild-type N2, mutants of *daf-16(mu86)*, *skn-1(zu67)*, and *mdt-15(tm2182)*, and transgenic strain of *oxIs12*[Ex(P*unc-47::GFP*)], originally obtained from *Caenorhabditis* Genetics Center. They were maintained on nematode growth medium (NGM) plates seeded with *Escherichia coli* OP50 at 20°C as described [79]. Age synchronous populations of L1- or L4-larvae were obtained as described previously [80]. Exposures were performed from L1-larvae to young adult in 12-well sterile tissue culture plates at 20°C in the presence of food. Exposed nematodes were used for toxicity assessment of GF with lethality, lifespan, growth, brood size, locomotion behavior, defecation, and intestinal ROS production as the endpoints.

### Lethality and Growth

For lethality assay, a 1.0 mL aliquot of test solution was added to each well of tissue culture plate, which was subsequently loaded with 50 nematodes for each treatment. Following exposure, wells were observed under a dissecting microscopy, where inactive ones were scored. Nematodes were judged to be dead if they did not respond to stimulus using a small, metal wire.

Growth was assessed by the body length, which was determined by measuring flat surface area of nematodes using Image-Pro® Express software. Twenty nematodes were examined per treatment.

### Lifespan Assay

In the test, the hermaphrodites were transferred daily for the first 4 days of adulthood. Nematodes were checked every day and would be scored as dead when they did not move even after repeated taps with a pick. Thirty nematodes were examined per treatment. For lifespan, graphs are representative of three trials.

### Reproduction and Locomotion Behavior

Reproduction was assessed by the brood size. To assay brood size, the number of offspring at all stages beyond the egg was counted. Twenty nematodes were examined per treatment.

Locomotion behavior was evaluated by both the head thrash and the body bend. To assay head thrash, the examined nematodes were washed with K medium, and transferred into a microtiter well containing 60 µL of K medium on the top of agar. After a 1-min recovery period, head thrash, defined as a change in the direction of bending at the mid body, was counted for 1-min. To assay body bend, the examined nematodes were picked onto a second plate and scored for the number of body bends in an interval of 20-sec. A body bend was counted as a change in the direction of the part of nematodes corresponding to the posterior bulb of the pharynx along the *y-*axis, assuming that nematode was traveling along the *x-*axis. Thirty nematodes were examined per treatment.

### Defecation

To assay mean defecation cycle length, individual animal was examined for a fixed number of cycles, and a cycle period was defined as the interval between initiations of two successive posterior body-wall muscle contraction steps. Thirty nematodes were examined per treatment.

### Intestinal ROS Production

The examined nematodes were transferred to 1 mL of M9 buffer containing 1 µM 5′,6′-chloromethyl-2′,7′dichlorodihydro-fluorescein diacetate (CM-H_2_DCFDA; Molecular Probes) in 12-well sterile tissue culture plates to pre-incubate for 3-hr at 20°C in the dark. CM-H_2_DCFDA can specially detect the presence of various intracellular produced ROS species. The intracellular ROS can oxidate the H_2_DCF without fluorescence to generate the DCF with green fluorescence. The nematodes were then mounted on 2% agar pads for examination with a laser scanning confocal microscope (Leica, TCS SP2, Bensheim, Germany) at 488 nm of excitation wavelength and 510 nm of emission filter. The relative fluorescent intensities of intestines were semi-quantified, and the semiquantified ROS was expressed as relative fluorescent units (RFU). Twenty nematodes were examined per treatment.

### Quantitative Real-time Polymerase Chain Reaction (PCR)

Total RNA was extracted using RNeasy Mini Kit (Qiagen). Total nematode RNA (∼1 µg) was reverse-transcribed using cDNA synthesis kit (Bio-Rad Laboratories). Quantitative reverse transcription PCR was run at the optimized annealing temperature of 58°C. The relative quantification of targeted genes in comparison to the reference *tba-1* gene encoding the tubulin protein or *act-1* gene encoding the actin protein was determined. The final results were expressed as the relative expression ratio (between targeted gene and reference gene). The designed primers for targeted genes and reference *tba-1* or *act-1* gene were shown in Table S2.

### Analysis of the Axonal Degeneration and Neuronal Loss of D-type GABAergic Motor Neurons

The method was performed as described [81]. D-type GABAergic motor neurons were visualized using a transgenic strain of *oxIs12* [82]. Gap numbers of ventral or dorsal cord were quantified to reflect the axonal degeneration. Neuronal loss was examined by counting the number of cell bodies in D-type GABAergic nervous system. Images were photographed and examined on the same day to avoid effects of light source variance on fluorescent intensity. Thirty nematodes were examined per treatment.

### Fluorescent Images of Neurons Controlling Defecation Behavior

The fluorescent images of AVL and DVB neurons controlling defecation behavior were captured with a Zeiss Axiocam MRm camera on a Zeiss Axioplan 2 Imaging System using SlideBook software (Intelligent Imaging Innovations). Images were acquired with a Quantix cooled charge-coupled device (CCD) camera, and illumination was provided by a 175 W xenon arc lamp and GFP filter sets. The relative sizes of fluorescent puncta for cell bodies of AVL and DVB neurons were measured as the maximum radius for assayed fluorescent puncta. Twenty nematodes were examined per treatment.

### Analysis of Chemical Components of Raw-processed GF

Before analysis of chemical components of GF, we added 600 µL methanol to 200 µL prepared GF, and then the suspensions were centrifugated for 10-min at 13000 rpm. The RP-HPLC method was applied to establish the chromatographic fingerprint. The separation was performed on a Elite C18 column with a mobile phase composed of methanol (A) and 0.25% acetic acid (B). Gradient elution condition was 0-min, 20% (A); 30-min, 60% (A); 45-min, 100% (A); and 50-min, 20% (A). The column temperature was set at 30°C and the flow rate was 1.0 mL/min. The detective wavelength was set at 260 nm. The injection volume was 10 µL. According to the HPLC data, the main components of prepared GF were determined.

### Statistical Analysis

All data in this article were expressed as means ± standard error of the mean (S.E.M.). Graphs were generated using Microsoft Excel (Microsoft Corp., Redmond, WA). Statistical analysis was performed using SPSS 12.0 (SPSS Inc., Chicago, USA). Differences between groups were determined using analysis of variance (ANOVA). Probability levels of 0.05 and 0.01 were considered statistically significant.

## Supporting Information

Figure S1 **Prolonged exposure to GF did not induce lethality in nematodes.** (A) Schematic drawing showing the prolonged exposure to GF. (B) Effects of GF exposure on lethality. GF, Genkwa Flos. Exposures were performed from L1-larvae to young adult. Bars represent means ± S.E.M.
(DOC)

Figure S2 **Expression patterns of genes required for intestinal development in control and GF exposed nematodes.** The results were expressed as the relative expression ratio between the targeted gene and the reference *act-1* gene. GF, Genkwa Flos. Exposures were performed from L1-larvae to young adult. Bars represent means ± S.E.M. **p<0.01.
(DOC)

Table S1 **Information on genes required for intestinal development in** ***C. elegans.***
(DOC)

Table S2 **Primers used for quantitative real-time polymerase chain reaction (PCR).**
(DOC)

## References

[1] Jiangsu New Medical College (1985) The encyclopedia of traditional Chinese medicine. 2nd ed., Shanghai Science and Technology Press, Shanghai.

[2] Wang R, Li J, Qi H, Shi Y (2013) Two new tigliane diterpene esters from the flower buds of *Daphne genkwa* . J Asian Nat Produt Res 15: 502–506. 10.1080/10286020.2013.786703 23600859

[3] Xie H, Liang Y, Ito Y, Wang X, Chen R, et al (2011) Preperative isolation and purification of four flavonoids from *Daphne genkwa* Sieb. et Zucc. by high-speed countercurrent chromatography. J Liq Chromatogr Relat Technol 34: 2360–2372. 22379361 10.1080/10826076.2011.589094 PMC3286800

[4] Lee M, Yuk J, Kwon O, Oh S, Lee H, et al (2012) Zuonin B inhibits lipopolysaccharide-induced inflammation via downregulation of the ERK1/2 and JNK pathways in RAW264.7 macrophages. Evid-Based Compl Alt Med 2012: 728196. 10.1155/2012/728196 PMC3292044 22454678

[5] Park B, Min B, Oh S, Kim J, Bae K, et al (2006) Isolation of flavonoids, a biscoumarin and an amide from the flower buds of *Daphne genkwa* and the evaluation of their anti-complement activity. Phytother Res 20: 610–613. 16685682 10.1002/ptr.1915

[6] Bang KK, Yun C, Lee C, Jin Q, Lee JW, et al (2013) Melanogenesis inhibitory daphnane diterpenoids from the flower buds of *Daphne genkwa* . Bioorg Med Chem Lett 23: 3334–3337. 23623417 10.1016/j.bmcl.2013.03.096

[7] Chang C, Leu Y, Horng J (2012) *Daphne genkwa* Sieb. et Zucc. water-soluble extracts act on enterovirus 71 by inhibiting viral entry. Viruses 4: 539–556. 22590685 10.3390/v4040539 PMC3347322

[8] Park B, Oh S, Ahn K, Kwon O, Lee H (2008) (-)-syringaresinol inhibits proliferation of human promyelocytic HL-60 leukemia cells via G1 arrest and apoptosis. Int Immunopharmacol 8: 967–973. 18486907 10.1016/j.intimp.2008.02.012

[9] Yang CL (1993) Toxic herbal medicine. Traditional Chinese Medicine Press, Beijing.

[10] Zhang S, Li X, Zhang F, Yang P, Gao X, et al (2006) Preparation of yuanhuacine and relative diterpene esters from *Daphne genkwa* and structure-activity relationship of potent inhibitory activity against DNA topoisomerase I. Bioorg Med Chem. 14: 3888–3895. 10.1016/j.bmc.2006.01.055 16488610

[11] Zheng W, Gao X, Chen C, Tan R (2007) Total flavonoids of *Daphne genkwa* root significantly inhibit the growth and metastasis of Lewis lung carcinoma in C57BL6 mice. Int Immunopharmacol 7: 117–127. 17178377 10.1016/j.intimp.2006.07.010

[12] Zheng W, Gao X, Gu Q, Chen C, Wei Z, et al (2007) Antitumor activity of daphnodorins from *Daphne genkwa* roots. Int Immunopharmacol 7: 128–134. 17178378 10.1016/j.intimp.2006.07.011

[13] Hong J, Nam J, Seo E, Lee S (2010) Daphnane diterpene esters with anti-proliferative activities against human lung cancer cells from *Daphne genkwa* . Chem Pharm Bull 58: 234–237. 20118586 10.1248/cpb.58.234

[14] Hong J, Chung H, Lee H, Park H, Lee S (2011) Growth inhibition of human lung cancer cells via down-regulation of epidermal growth factor receptor signaling by Yuanhuadine, a daphnane diterpene from *Daphne genkwa* . J Nat Prod 74: 2102–2108. 21916433 10.1021/np2003512

[15] Li F, Sun Q, Hong L, Li L, Wu Y, et al (2013) Daphnane-type diterpenes with inhibitory activities against human cancer cell lines from *Daphne genkwa* . Bioorg Med Chem Lett 23: 2500–2504. 23558238 10.1016/j.bmcl.2013.03.025

[16] Zhan Z, Fan C, Ding J, Yue J (2005) Novel diterpenoids with potent inhibitory activity against endothelium cell HMEC and cytotoxic activities from a well-known TCM plant *Daphne genkwa* . Biorag Med Chem 13: 645–655. 10.1016/j.bmc.2004.10.054 15653331

[17] Geng L, Ma C, Zhang L, Yang G, Cui Y, et al (2013) Metabonomic study of Genkwa Flos-induced hepatotoxicity and effect of herb-processing procedure on toxicity. Phytother Res 27: 521–529. 22648611 10.1002/ptr.4748

[18] Li Z, Li Q, Geng L, Chen X, Bi K (2013) Use of the local false discovery rate for identififcation of metabolic biomarkers in rat urine following Genkwa Flos-induced hepatotoxicity. PLoS ONE 8: e67451. 23844011 10.1371/journal.pone.0067451 PMC3699555

[19] Chen Y, Guo J, Qian Y, Guo S, Ma C, et al (2013) Toxicity of daphnane-type diterpenoids from Genkwa Flos and their pharmacokinetic profile in rat. Phytomedicine 21: 82–89. 23988178 10.1016/j.phymed.2013.06.012

[20] Leung MCK, Williams PL, Benedetto A, Au C, Helmcke KJ, et al (2008) *Caenorhabditis elegans*: an emerging model in biomedical and environmental toxicology. Toxicol Sci 106: 5–28. 18566021 10.1093/toxsci/kfn121 PMC2563142

[21] Zhao Y–L, Wang D–Y (2012) Formation and regulation of adaptive response in nematode *Caenorhabditis elegans* . Oxid Med Cell Longev 2012: 564093. 22997543 10.1155/2012/564093 PMC3446806

[22] ZhaoY–L, Wu Q–L, Li Y–P, Wang D–Y (2013) Translocation, transfer, and *in vivo* safety evaluation of engineered nanomaterials in the non-mammalian alternative toxicity assay model of nematode *Caenorhabditis elegans* . RSC Adv 3: 5741–5757.

[23] Hu Y-O, Wang Y, Ye B-P, Wang D-Y (2008) Phenotypic and behavioral defects induced by iron exposure can be transferred to progeny in *Caenorhabditis elegans* . Biomed Environ Sci 21: 467–473. 19263801 10.1016/S0895-3988(09)60004-0

[24] Xing X-J, Du M, Zhang Y-F, Wang D-Y (2009) Adverse effects of metal exposure on chemotaxis towards water-soluble attractants regulated mainly by ASE sensory neuron in nematode *Caenorhabditis elegans* . J Environ Sci 21: 1684–1694. 10.1016/s1001-0742(08)62474-2 20131599

[25] Xing X-J, Wang D-Y (2009) The lethality toxicities induced by metal exposure during development in nematode *Caenorhabditis elegans* . Bull Environ Contam Toxicol 83: 530–536. 19588066 10.1007/s00128-009-9816-3

[26] Wang D-Y, Xing X-J (2009) Pre-treatment with mild metal exposure suppresses the neurotoxicity on locomotion behavior induced by the subsequent severe metal exposure in *Caenorhabditis elegans* . Environ Toxicol Pharmacol 28: 459–464. 21784043 10.1016/j.etap.2009.07.008

[27] Helmcke KJ, Syversen T, Miller III DM, Aschner M (2009) Characterization of the effects of methylmercury on *Caenorhabditis elegans* . Toxicol Appl Pharmacol 240: 265–272. 19341752 10.1016/j.taap.2009.03.013 PMC2753706

[28] Wang S, Wu L, Wang Y, Luo X, Lu Y (2009) Copper-induced germline apoptosis in *Caenorhabditis elegans*: the independent roles of DNA damage response signaling and the dependent roles of MAPK cascades. Chemico-Biol Interactions 180: 151–157. 10.1016/j.cbi.2009.03.012 19497412

[29] Wang D-Y, Xing X-J (2010) Pre-treatment with mild UV irradiation suppresses reproductive toxicity induced by subsequent cadmium exposure in nematodes. Ecotoxicol Environ Safety 73: 423–429. 20045190 10.1016/j.ecoenv.2009.12.014

[30] Wang D-Y, Liu P-D, Yang Y-C, Shen L-L (2010) Formation of combined Ca/Cd toxicity on lifespan of nematode *Caenorhabditis elegans* . Ecotoxicol Environ Safety 73: 1221–1230. 20580433 10.1016/j.ecoenv.2010.05.002

[31] Zeitoun-Ghandour S, Charnock JM, Hodson ME, Leszczyszyn OI, Blindauer CA, et al (2010) The two *Caenorhabditis elegans* metallothioneins (CeMT-1 and CeMT-2) discriminate between essential zinc and toxic cadmium. FEBS J 277: 2531–2542. 20553489 10.1111/j.1742-4658.2010.07667.x

[32] Zhang H, He X, Bai W, Guo X, Zhang Z, et al (2010) Ecotoxicological assessment of lanthanum with *Caenorhabditis elegans* in liquid medium. Metallomics 2: 806–810. 21510015 10.1039/c0mt00059k

[33] Wu Q-L, He K-W, Liu P-D, Li Y-X, Wang D-Y (2011) Association of oxidative stress with the formation of reproductive toxicity from mercury exposure on hermaphrodite nematode *Caenorhabditis elegans.* . Environ Toxicol Pharmacol 32: 175–184. 21843797 10.1016/j.etap.2011.04.009

[34] Jiang B, Ren C, Li Y, Lu Y, Li W, et al (2011) Sodium sulfite is a potential hypoxia inducer that mimics hypoxic stress in *Caenorhabditis elegans* . J Biol Inorg Chem 16: 267–274. 21057967 10.1007/s00775-010-0723-1

[35] Liu P-D, He K-W, Li Y-X, Wu Q-L, Yang P, et al (2012) Exposure to mercury causes formation of male-specific structural deficits by inducing oxidative damage in nematodes. Ecotoxicol Environ Safety 79: 90–100. 22209111 10.1016/j.ecoenv.2011.12.007

[36] Wu Q-L, Qu Y-Y, Li X, Wang D-Y (2012) Chromium exhibits adverse effects at environmental relevant concentrations in chronic toxicity assay system of nematode *Caenorhabditis elegans* . Chemosphere 87: 1281–1287. 22336735 10.1016/j.chemosphere.2012.01.035

[37] Li Y-H, Ye H-Y, Du M, Zhang Y-F, Ye B-P, et al (2009) Induction of chemotaxis to sodium chloride and diacetyl and thermotaxis defects by microcystin-LR exposure in nematode *Caenorhabditis elegans* . J Environ Sci 21: 971–979. 10.1016/s1001-0742(08)62370-0 19862965

[38] Ruan Q-L, Ju J-J, Li Y-H, Liu R, Pu Y-P, et al (2009) Evaluation of pesticide toxicities with differing mechanisms using *Caenorhabditis elegans* . J Toxicol Environ Health A 72: 746–751. 19492238 10.1080/15287390902841532

[39] Boyd WA, Smith MV, Kissling GE, Rice JR, Snyder DW, et al (2009) Application of mathematical model to describe the effects of chlorpyrifos on *Caenorhabditis elegans* development. PLoS ONE 4: e7024. 19753116 10.1371/journal.pone.0007024 PMC2737145

[40] Allard P, Colaiacovo MP (2010) Bisphenol A impairs the double-strand break repair machinery in the germline and causes chromosome abnormalities. Proc Natl Acad Sci USA 107: 20405–20410. 21059909 10.1073/pnas.1010386107 PMC2996676

[41] Ruan Q-L, Ju J-J, Li Y-H, Li X-B, Liu R, et al (2012) Chlorpyrifos exposure reduces reproductive capacity owing to a damaging effect on gametogenesis in the nematode *Caenorhabditis elegans* . J Appl Toxicol 32: 527–535. 22180373 10.1002/jat.1783

[42] Ju J-J, Ruan Q-L, Li X-B, Liu R, Li Y-H, et al (2013) Neurotoxicological evaluation of microcystin-LR exposure at environmental relevant concentrations on nematode *Caenorhabditis elegans* . Environ Sci Pollut Res 20: 1823–1830. 10.1007/s11356-012-1151-2 22956115

[43] Ma H, Bertsch PM, Glenn TC, Kabengi NJ, Williams PL (2009) Toxicity of manufactured zinc oxide nanoparticles in the nematode *Caenorhabditis elegans* . Environ Toxicol Chem 28: 1324–1330. 19192952 10.1897/08-262.1

[44] Mohan N, Chen C, Hsieh H, Wu Y, Chang H (2010) In vivo imaging and toxicity assessments of fluorescent nanodiamonds in *Caenorhabditis elegans* . Nano Lett 10: 3692–3699. 20677785 10.1021/nl1021909

[45] Qu Y, Li W, Zhou Y, Liu X, Zhang L, et al (2011) Full assessment of fate and physiological behavior of quantum dots utilizing *Caenorhabditis elegans* as a model organism. Nano Lett 11: 3174–3183. 21721562 10.1021/nl201391e

[46] Yu S-H, Rui Q, Cai T, Wu Q-L, Li Y-X, et al (2011) Close association of intestinal autofluorescence with the formation of severe oxidative damage in intestine of nematodes chronically exposed to Al_2_O_3_-nanoparticle. Environ Toxicol Pharmacol 32: 233–241. 21843804 10.1016/j.etap.2011.05.008

[47] Yang X, Gondikas AP, Marinakos SM, Auffan M, Liu J, et al (2012) Mechanism of silver nanoparticle toxicity is dependent in dissolved silver and surface coating in *Caenorhabditis elegans* . Environ Sci Technol 46: 1119–1127. 22148238 10.1021/es202417t

[48] Wu Q-L, Li Y-P, Tang M, Wang D-Y (2012) Evaluation of environmental safety concentrations of DMSA coated Fe_2_O_3_-NPs using different assay systems in nematode *Caenorhabditis elegans* . PLoS ONE 7: e43729. 22912902 10.1371/journal.pone.0043729 PMC3422352

[49] Li Y-X, Wang W, Wu Q-L, Li Y-P, Tang M, et al (2012) Molecular control of TiO_2_-NPs toxicity formation at predicted environmental relevant concentrations by Mn-SODs proteins. PLoS ONE 7: e44688. 22973466 10.1371/journal.pone.0044688 PMC3433426

[50] Wu Q-L, Wang W, Li Y-X, Ye B-P, Tang M, et al (2012) Small sizes of TiO_2_-NPs exhibit adverse effects at predicted environmental relevant concentrations on nematodes in a modified chronic toxicity assay system. J Hazard Mater 243: 161–168. 23127274 10.1016/j.jhazmat.2012.10.013

[51] Li Y-X, Yu S-H, Wu Q-L, Tang M, Wang D-Y (2013) Transmissions of serotonin, dopamine and glutamate are required for the formation of neurotoxicity from Al_2_O_3_-NPs in nematode *Caenorhabditis elegans* . Nanotoxicology 7: 1004–1013. 22548316 10.3109/17435390.2012.689884

[52] Zhao Y-L, Wu Q-L, Tang M, Wang D-Y (2014) The *in vivo* underlying mechanism for recovery response formation in nano-titanium dioxide exposed *Caenorhabditis elegans* after transfer to the normal condition. Nanomedicine: Nanotechnol Biol Med 10: 89–98. 10.1016/j.nano.2013.07.004 23891985

[53] Rui Q, Zhao Y-L, Wu Q-L, Tang M, Wang D-Y (2013) Biosafety assessment of titanium dioxide nanoparticles in acutely exposed nematode *Caenorhabditis elegans* with mutations of genes required for oxidative stress or stress response. Chemosphere 93: 2289–2296. 24001673 10.1016/j.chemosphere.2013.08.007

[54] Ye H-Y, Ye B-P, Wang D-Y (2008) Trace administration of vitamin E can retrieve and prevent UV-irradiation- and metal exposure-induced memory deficits in nematode *Caenorhabditis elegans* . Neurobiol Learn Mem 90: 10–18. 18191592 10.1016/j.nlm.2007.12.001

[55] Onken B, Driscoll M (2010) Metformin induces a dietary restriction-like state and the oxidative stress response to extend *C. elegans* healthspan via AMPK, LKB1, and SKN-1. PLoS ONE 5: e8758. 20090912 10.1371/journal.pone.0008758 PMC2807458

[56] Kumar S, Aninat C, Michaux G, Morel F (2010) Anticancer drug 5-fluorouracil induces reproductive and developmental defects in *Caenorhabditis elegans* . Reprod Toxicol 29: 415–420. 20206682 10.1016/j.reprotox.2010.02.006

[57] Lublin A, Isoda F, Patel H, Yen K, Ngugen L, et al (2011) FDA-approved drugs that protect mammalian neurons from glucose toxicity slow aging dependent on cbp and protect against proteotoxicity. PLoS ONE 6: e27762. 22114686 10.1371/journal.pone.0027762 PMC3218048

[58] Li Y-P, Li Y-X, Wu Q-L, Ye H-Y, Sun L-M, et al (2013) High concentration of vitamin E decreases thermosensation and thermotaxis learning and the underlying mechanisms in nematode *Caenorhabditis elegans* . PLoS ONE 8: e71180. 23951104 10.1371/journal.pone.0071180 PMC3741368

[59] Rui Q, Lu Q, Wang DY (2009) Administration of *Bushenkangshuai Tang* alleviates the UV irradiation- and oxidative stress-induced lifespan defects in nematode *Caenorhabditis elegans* . Front Med China 3: 76–90.

[60] Dostal V, Roberts CM, Link CD (2010) Genetic mechanisms of coffee extract protection in a *Caenorhabditis elegans* model of β-amyloid peptide toxicity. Genetics 186: 857–866. 20805557 10.1534/genetics.110.120436 PMC2975290

[61] Zhang W-M, Lv T, Li M, Wu Q-L, Yang L-S, et al (2013) Beneficial effects of wheat gluten hydrolysate to extend lifespan and induce stress resistance in nematode *Caenorhabditis elegans* . PLoS ONE 8: e74553. 24040279 10.1371/journal.pone.0074553 PMC3767650

[62] Mukai D, Matsuda N, Yoshioka Y, Sato M, Yamasaki T (2008) Potential anthelmintics : polyphenols from the tea plant *Camellia sinensis* L. are lethally toxic to *Caenorhabditis elegans* . J Nat Med 62: 155–159. 18404315 10.1007/s11418-007-0201-4

[63] Fan D, Hodges DM, Zhang J, Kirby CW, Ji X, et al (2011) Commercial extract of the brown seaweed *Ascophyllum nodosum* enhances phenolic antioxidant contents of spinach (*Spinacia oleracea* L.) which protects *Caenorhabditis elegans* against oxidative and thermal stress. Food Chem 124: 195–202.

[64] Sangha JS, Sun X, Wally OSD, Zhang K, Ji X, et al (2012) Liuwei Dihuang (LWDH), a traditional Chinese medicinal formula, protects against β-amyloid toxicity in transgenic *Caenorhabditis elegans* . PLoS ONE 7: e43990. 22952840 10.1371/journal.pone.0043990 PMC3431378

[65] Dharmalingam K, Yan B, Mahmud MZ, Sedek SAM, Majid MIA, et al (2012) *Swietenia macrophylla* extract promotes the ability of *Caenorhabditis elegans* to survive *Pseudomonas aeruginosa* infection. J Ethnopharmacol 139: 657–663. 22193176 10.1016/j.jep.2011.12.016

[66] Kandasamy S, Khan W, Evans F, Critchley AT, Prithiviraj B (2012) Tasco®: a product of *Ascophyllum nodosum* enhances immune response of *Caenorhabditis elegans* against *Pseudomonas aeruginosa* infection. Mar Drugs 10: 84–105. 22363222 10.3390/md10010084 PMC3280538

[67] Wu Q-L, Li Y-X, Li Y-P, Zhao Y-L, Ge L, et al (2013) Crucial role of biological barrier at the primary targeted organs in controlling translocation and toxicity of multi-walled carbon nanotubes in nematode *Caenorhabditis elegans.* . Nanoscale 5: 11166–11178. 24084889 10.1039/c3nr03917j

[68] Wu Q-L, Yin L, Li X, Tang M, Zhang T, et al (2013) Contributions of altered permeability of intestinal barrier and defecation behavior to toxicity formation from graphene oxide in nematode *Caenorhabditis elegans* . Nanoscale 5: 9934–9943. 23986404 10.1039/c3nr02084c

[69] Nouara A, Wu Q-L, Li Y-X, Tang M, Wang H-F, et al (2013) Carboxylic acid functionalization prevents the translocation of multi-walled carbon nanotubes at predicted environmental relevant concentrations into targeted organs of nematode *Caenorhabditis elegans* . Nanoscale 5: 6088–6096. 23722228 10.1039/c3nr00847a

[70] Li Y-X, Yu S-H, Wu Q-L, Tang M, Pu Y-P, et al (2012) Chronic Al_2_O_3_-nanoparticle exposure causes neurotoxic effects on locomotion behaviors by inducing severe ROS production and disruption of ROS defense mechanisms in nematode *Caenorhabditis elegans* . J Hazard Mater 219–220: 221–230. 10.1016/j.jhazmat.2012.03.083 22521136

[71] McIntire SL, Jorgensen E, Kaplan J, Horvitz HR (1993) The GABAergic nervous system of *Caenorhabditis elegans* . Nature 364: 337–341. 8332191 10.1038/364337a0

[72] Pluskota A, Horzowski E, Bossinger O, von Mikecz A (2009) In *Caenorhabditis elegans* nanoparticle-bio-interactions become transparent: silica-nanoparticles induce reproductive senescence. PLoS ONE 4: e6622. 19672302 10.1371/journal.pone.0006622 PMC2719910

[73] McGhee JD (2007) The *C. elegans* intestine. WormBook, ed. The *C. elegans* Research Community, WormBook, doi: 10.1895/wormbook.1.133.1.

[74] Lapierre LR, Hansen M (2012) Lessons from *C. elegans* : signaling pathways for longevity. Trend Endocrinol Metab 23: 637–644. 10.1016/j.tem.2012.07.007 PMC3502657 22939742

[75] Tullet JM, Hertweck M, An JH, Baker J, Hwang JY, et al (2008) Direct inhibition of the longevity-promoting factor SKN-1 by insulin-like signaling in *C. elegans* . Cell 132: 025–1038. 10.1016/j.cell.2008.01.030 PMC2367249 18358814

[76] Goh GY, Martelli KL, Parhar KS, Kwong AW, Wong MA, et al. (2013) The conserved mediator subunit MDT-15 is required for oxidative stress responses in *Caenorhabditis elegans*. Aging Cell doi: 10.1111/acel.12. 10.1111/acel.12154 PMC4326869 23957350

[77] Wu S, Lu J-H, Rui Q, Yu S-H, Cai T, et al (2011) Aluminum nanoparticle exposure in L1 larvae results in more severe lethality toxicity than in L4 larvae or young adults by strengthening the formation of stress response and intestinal lipofuscin accumulation in nematodes. Environ Toxicol Pharmacol 31: 179–188. 21787684 10.1016/j.etap.2010.10.005

[78] Wang D-Y, Wang Y (2008) Phenotypic and behavioral defects caused by barium exposure in nematode *Caenorhabditis elegans* . Arch Environ Contam Toxicol 54: 447–453. 17932710 10.1007/s00244-007-9050-0

[79] Brenner S (1974) The genetics of *Caenorhabditis elegans* . Genetics 77: 71–94. 4366476 10.1093/genetics/77.1.71 PMC1213120

[80] Donkin S, Williams PL (1995) Influence of developmental stage, salts and food presence on various end points using *Caenorhabditis elegans* for aquatic toxicity testing. Environ Toxicol Chem 14: 2139–2147.

[81] Williams PL, Dusenbery DB (1990) Aquatic toxicity testing using the nematode *Caenorhabditis elegans* . Environ Toxicol Chem 9: 1285–1290. 11345460

[82] Du M, Wang D-Y (2009) The neurotoxic effects of heavy metal exposure on GABAergic system in nematode *Caenorhabditis elegans* . Environ Toxicol Pharmacol 27: 314–320. 21783959 10.1016/j.etap.2008.11.011

