# Supplementary material for: Full Toxicity Assessment of Genkwa Flos and the Underlying Mechanism in Nematode Caenorhabditis elegans
Source: PLoS One. 2014 Mar 13;9(3):e91825. doi: 10.1371/journal.pone.0091825 (PMC3953530; doi:10.1371/journal.pone.0091825)
Supplement: Figure S1 — Prolonged exposure to GF did not induce lethality in nematodes. (A) Schematic drawing showing the prolonged exposure to GF. (B) Effects of GF exposure on lethality. GF, Genkwa Flos. Exposures were performed from L1-larvae to young adult. Bars represent means ± S.E.M. (DOC) [file pone.0091825.s001.doc]

**
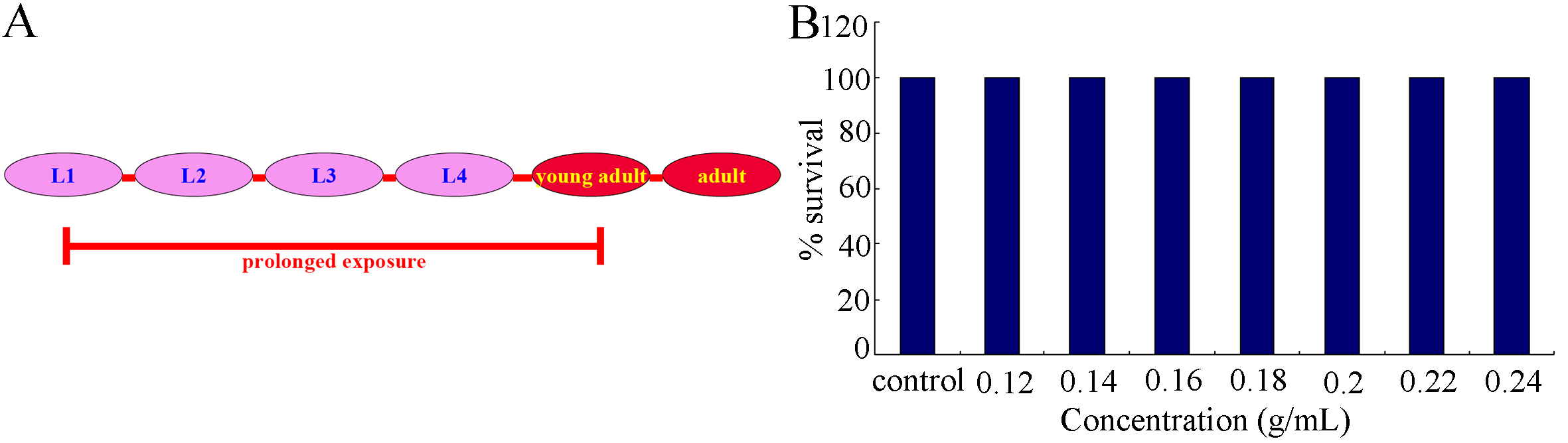
**

**Figure S1. Prolonged exposure to GF did not induce lethality in nematodes.** (A) Schematic drawing showing the prolonged exposure to GF. (B) Effects of GF exposure on lethality. GF, Genkwa Flos. Exposures were performed from L1-larvae to young adult. Bars represent means ± S.E.M.
