# Supplementary material for: Full Toxicity Assessment of Genkwa Flos and the Underlying Mechanism in Nematode Caenorhabditis elegans
Source: PLoS One. 2014 Mar 13;9(3):e91825. doi: 10.1371/journal.pone.0091825 (PMC3953530; doi:10.1371/journal.pone.0091825)
Supplement: Figure S2 — Expression patterns of genes required for intestinal development in control and GF exposed nematodes. The results were expressed as the relative expression ratio between the targeted gene and the reference act-1 gene. GF, Genkwa Flos. Exposures were performed from L1-larvae to young adult. Bars represent means ± S.E.M. **p<0.01. (DOC) [file pone.0091825.s002.doc]

**
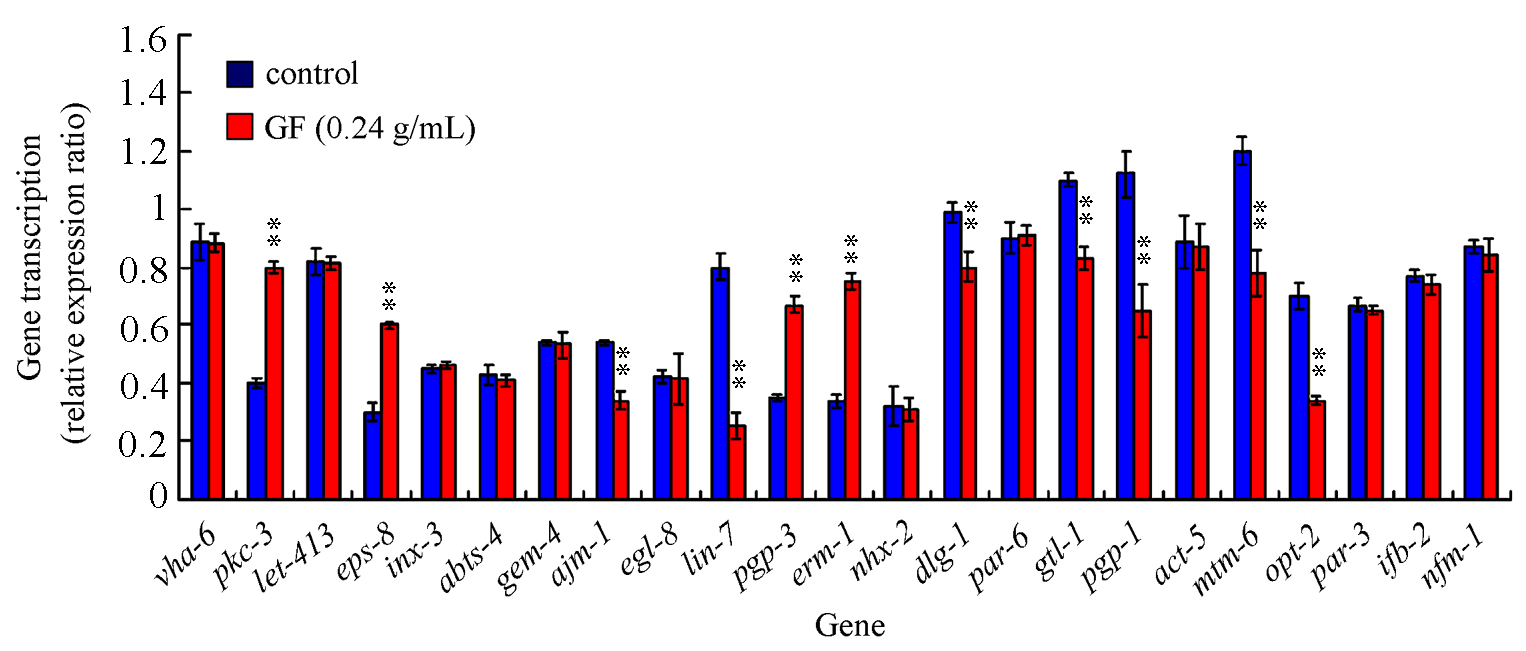
**

**Figure S2. Expression patterns of genes required for intestinal development in control and GF exposed nematodes.**  The results were expressed as the relative expression ratio between the targeted gene and the reference *act-1* gene. GF, Genkwa Flos. Exposures were performed from L1-larvae to young adult. Bars represent means ± S.E.M. ^**^*p* < 0.01.
