# Supplementary material for: Full Toxicity Assessment of Genkwa Flos and the Underlying Mechanism in Nematode Caenorhabditis elegans
Source: PLoS One. 2014 Mar 13;9(3):e91825. doi: 10.1371/journal.pone.0091825 (PMC3953530; doi:10.1371/journal.pone.0091825)
Supplement: Table S1 — Information on genes required for intestinal development in C. elegans. (DOC) [file pone.0091825.s003.doc]

**Table S1. Information on genes required for intestinal development in *C. elegans***

| Gene | Products of the genes |
| --- | --- |
| *gem-4* | Ca^2+^-dependent phosphatidylserine binding protein |
| *mtm-6* | myotubularin lipid phosphatase orthologous |
| *nhx-2* | sodium/proton exchanger |
| *opt-2* | high-affinity, proton-coupled oligopeptide transporter |
| *pkc-3* | atypical protein kinase |
| *par-3* | PDZ domain-containing protein orthologous |
| *par-6* | PDZ-domain-containing protein |
| *pgp-1* | transmembrane protein |
| *pgp-3* | transmembrane protein |
| *vha-6* | membrane-bound (V0) domain of vacuolar proton-translocating ATPase (V-ATPase); |
| *gtl-1* | TRPM subfamily member of the TRP channel family |
| *erm-1* | ortholog of the ERM family of cytoskeletal linkers |
| *eps-8* | homolog of mouse epidermal growth factor receptor kinase substrate |
| *act-5* | ortholog of human cytoplasmic actin |
| *ifb-2* | nonessential intermediate filament protein |
| *dlg-1* | MAGUK protein |
| *ajm-1* | member of the apical junction molecule class |
| *egl-8* | phospholipase C beta homolog |
| *let-413* | protein with strong similarity to human ERBIN, rat DENSIN, Drosophila SCRIB and its human ortholog hSCRIB |
| *nfm-1* | homolog of human merlin/schwannomin (NF2) |
| *inx-3* | gap protein |
| *lin-7* | a protein containing a PDZ domain and an L27 domain |
| *abts-4* | anion transporter |
