# Supplementary material for: Full Toxicity Assessment of Genkwa Flos and the Underlying Mechanism in Nematode Caenorhabditis elegans
Source: PLoS One. 2014 Mar 13;9(3):e91825. doi: 10.1371/journal.pone.0091825 (PMC3953530; doi:10.1371/journal.pone.0091825)
Supplement: Table S2 — Primers used for quantitative real-time polymerase chain reaction (PCR). (DOC) [file pone.0091825.s004.doc]

**Table S2. Primers used for** quantitative real-time polymerase chain reaction (PCR)

| Gene | Forward primer | Reverse primer |
| --- | --- | --- |
| *act-1* | CTGCAGATGTGTGACGACGAGGTT | CTGCAGGAAGCACTTGCGGTGAAC |
| *tba-1* | TCAACACTGCCATCGCCGCC | TCCAAGCGAGACCAGGCTTCAG |
| *gem-4* | CACGGTGGTCAACAGTAT | TTGTATTTGGCACCTTTC |
| *mtm-6* | AAAAGGGACGCTAACAGC | ATTCTCAAACGCAAGCAG |
| *nhx-2* | GGAGCAGAATGTGAAGAA | GTGGCGGAAGTAGATAAA |
| *opt-2* | ACTGGTATTTATGGGAGGTT | AAGAACACGGAGTAGGGA |
| *pho-1* | ACGGACATGATGTAGGAG | ATTAGAAGTGCGGAGAAG |
| *pkc-3* | CGTCTCCGACATCATTAG | CAACTCGGCTTCTTGACT |
| *par-3* | AAGCGTAACTGTCAACCA | CCGTCTATAACATCCTCC |
| *par-6* | ATTCTGCGTCTGGTGTCT | TTCCCTTCCATCGTTTAT |
| *pgp-1* | AATGTCCGATTCGCTTAC | CTCAGGGTTCAACGTCTT |
| *pgp-3* | GGACTTCCTGACGGTTAC | TTTGATGGGTTCCTTCTT |
| *vha-6* | ATGGAGGCAAACTTAGAG | TTCCGAGATTGACATAGC |
| *gtl-1* | CTGCTCACCACGCACAAT | AACTCCTTCATCCAACCC |
| *erm-1* | TCCACGACTCCGTATCAA | TCCTGCTCGGCAATCTTA |
| *eps-8* | ACGCAGTGACGGTAGAAG | AGCGGATACACGGATACA |
| *act-5* | GGGAGTGATGGTCGGTAT | CGGTAAGGAGAACTGGGT |
| *ifb-2* | TCAAGGCTGAATACGACA | TCCAAAGCAGAGTTACGG |
| *dlg-1* | TTGAAACGGCGTAAAGAT | CGTGATGAACTGGTGGTG |
| *ajm-1* | GTCAATCAGTTCGTCCCG | ACTCGTCCGATGGTGTCT |
| *egl-8* | GCTCGATGGCTTCAAGTA | TGAATGCTATCCCTCTGC |
| *let-413* | TTGCGTCCAACAAGTTAC | CACCAAGAAATGCTCCTC |
| *nfm-1* | ATTACGGAGGATCTGGTA | TCATCGTCGTGAACTTAT |
| *inx-3* | CAGTGGGTGCCTATTGTG | GACCGTATTCGTTCTTGG |
| *abts-4* | CTCAGACTACAGGGATGG | GTGCCTGACTCACAAGAC |
| *lin-7* | GTTATGGGCGGCAAGGAG | CGTCGGGAGTGTTGGACT |
